# TRPV1 mediates mitochondrial dysfunction in hyperthermia–induced ameloblast mineralization defects

**DOI:** 10.3389/fcell.2026.1903038

**Published:** 2026-09-17

**Authors:** Jingyi Li, Jing Fu, Yiwen Pan, Huiwen Zheng, Xianghui Xing

**Affiliations:** Nanjing Stomatological Hospital, Affiliated Hospital of Medical School, Institute of Stomatology, Nanjing University, Nanjing, China

**Keywords:** ALCs, calcium homeostasis, enamel hypomineralization, hyperthermia, mitochondrial dysfunction, TRPV1

## Abstract

**Background:**

Enamel hypomineralization is a prevalent developmental dental defect associated with several early-life risk factors, including hyperthermia. However, the cellular mechanisms linking hyperthermia to impaired enamel mineralization remain unclear. This study investigated the potential role of transient receptor potential vanilloid 1 (TRPV1) in hyperthermia-associated ameloblast dysfunction.

**Methods:**

Using a mouse ameloblast-lineage cell (ALC) model, we performed quantitative real-time PCR (qRT‐PCR), alkaline phosphatase (ALP) staining, Alizarin Red S (ARS) staining, RNA sequencing, immunohistochemistry, Western blotting, molecular intervention experiments, intracellular and mitochondrial calcium imaging, reactive oxygen species measurements, and mitochondrial functional assays.

**Results:**

RNA sequencing and immunohistochemistry identified TRPV1 as a candidate heat-responsive channel expressed in ameloblasts. Hyperthermia was associated with reduced mineralization-related markers, increased intracellular and mitochondrial Ca^2+^ signals and reactive oxygen species levels, decreased mitochondrial membrane potential, and impaired ATP content and NAD^+^/NADH ratio. Pharmacological inhibition and genetic suppression of TRPV1 partially alleviated these hyperthermia-associated alterations.

**Conclusion:**

These findings support the involvement of TRPV1 in hyperthermia‐associated calcium dysregulation, mitochondrial dysfunction, and impaired mineralization-related responses in cultured ALCs. The results provide a testable framework for further in vivo investigation.

## 1 Introduction

Enamel hypomineralization is a developmental dental defect associated with multiple risk factors (Jälevik, 2010) and has become increasingly prevalent over recent years (Ammar et al., 2025). Previous studies have suggested that various factors such as environmental exposures can contribute to the pathogenesis of enamel developmental defects (Crombie et al., 2009; Whatling and Fearne, 2008; Juárez–López et al., 2023). Among these factors, thermal stress—such as hyperthermia during pregnancy or early tooth development in infants—is a common environmental factor (Weerheijm et al., 2003). Although the effects of heat stress on enamel have been reported previously (Ryynänen et al., 2014; Tung et al., 2006) (with a thermal threshold of approximately 39 °C reported *in vitro*), its underlying molecular mechanisms remain undefined.

Considering the pivotal role of ameloblasts in enamel mineralization (Malmberg et al., 2019), investigation of ameloblast activities may be crucial to understanding the underlying mechanisms. Ameloblasts directly determine the structural integrity and degree of mineralization of enamel (Malmberg et al., 2019; Smith et al., 2017; Lacruz et al., 2017). They mainly undergo three stages: the secretory, transition, and maturation (Crombie et al., 2009; Novacescu et al., 2025). Compared with the secretory stage, during which ameloblasts are primarily responsible for secretion of enamel matrix proteins (Simmer et al., 2010), mature ameloblasts mainly regulate ion transport essential for determining the final level of mineralization (Lacruz et al., 2012). Studies have demonstrated that intracellular calcium ions (Ca^2+^) transport and mitochondrial modulation are crucial for enamel mineralization (Walkon et al., 2022), contributing to the formation of a localized extracellular enamel microenvironment (Costiniti et al., 2020). However, excessive intracellular Ca^2+^ can impair enzymatic activity, disrupt mitochondrial cristae structure (Walkon et al., 2022), inhibit oxidative phosphorylation (Strubbe–Rivera et al., 2021), and ultimately impair mitochondrial bioenergetic function (Costiniti et al., 2022). Thus, maintaining Ca^2+^ homeostasis is vital for normal enamel formation (Nurbaeva et al., 2018). Dysregulation of Ca^2+^ homeostasis or mitochondrial dysfunction may compromise enamel formation, and in severe cases, may result in structural enamel lesions (Costiniti et al., 2021).

TRPV1, also known as the capsaicin receptor, is a key sensor for environmental noxious stimuli (Tominaga et al., 1998) such as hyperthermia, with an activation threshold ranging from 39 °C to 42 °C (Caterina et al., 1997; Garami et al., 2010). As a non–selective cation channel (Gao et al., 2024), TRPV1 serves as an important pathway for extracellular Ca^2+^ entry into cells. TRPV1 also modulates both intracellular and mitochondrial Ca^2+^ homeostasis (Juárez–Contreras et al., 2020). However, its excessive activation may cause mitochondrial membrane potential depolarization and impair biosynthetic processes (Tessier et al., 2023; Sun et al., 2014).

In summary, the mechanisms linking hyperthermia to enamel hypomineralization remain incompletely defined (Ryynänen et al., 2014; Tung et al., 2006). We therefore used ALCs to examine whether heat exposure impairs mineralization and whether TRPV1–associated calcium signaling and mitochondrial alterations contribute to this response. By combining ALP and ARS staining, qRT-PCR, RNA-seq, Western blotting, intracellular and mitochondrial Ca^2+^ measurements, and mitochondrial functional assays, we assessed the relationship among hyperthermia, TRPV1, calcium homeostasis, mitochondrial function, and mineralization–related readouts *in vitro*.

## 2 Materials and methods

### 2.1 Cell culture

ALCs were cultured in DMEM/F-12 medium (Gibco, USA) containing 10% FBS (Meilun Bio, China) and 1% penicillin–streptomycin (Gibco, USA) at 37 °C, 5% CO_2_, and saturated humidity. ALCs at the fifth passage were used for subsequent experiments.

### 2.2 Cell grouping and thermal treatment

The qRT-PCR experiment was used to screen the duration of hyperthermic treatment. ALCs were cultured for a total of 7 days under four mineralizing conditions: 37 °C for 7 days; 39 °C for 1 day followed by 37 °C for 6 days; 39 °C for 3 days followed by 37 °C for 4 days; and 39 °C for 5 days followed by 37 °C for 2 days. The temperature–treatment regimens used for the ALP and ARS staining experiments were selected based on the qRT-PCR results. Because detectable changes in ALP and ARS staining generally require a sufficient duration of treatment, the 39 °C 3‐days and 5‐days regimens were selected for these experiments. Cells were assigned to an unmineralized control (37 °C without mineralization induction), a mineralized control (37 °C), a 39 °C 3–days group, or a 39 °C 5 days group. All groups were maintained under otherwise identical culture conditions. The 39 °C condition was selected to model a clinically relevant high–fever temperature in children, to remain consistent with previous enamel–development studies (Barbi et al., 2017), and to fall within the lower reported range of heat sensitivity for TRPV1 (Caterina et al., 1997).

### 2.3 Cell osteogenic differentiation

When cells reached 50%–60% confluence, mineralization of ALCs was induced by replacing the medium with a mineralization–induction medium containing 50 mg/L ascorbic acid (Sigma, USA), 10 mM β–glycerophosphate sodium (Sigma, USA), and 10 nM dexamethasone (Sigma, USA). The medium was changed every 48 h, and the mineralization of the cells was observed each day.

### 2.4 ALP staining and ARS staining

To assess the effect of hyperthermia on ALC mineralization, ALP staining (day 7) and ARS staining (day 21) were performed after mineralization induction. Cells were fixed with 4% paraformaldehyde (Servicebio, China) for 15 min, washed with PBS, and stained using the BCIP/NBT ALP kit (Beyotime, China) or Alizarin Red S solution at 37 °C for 4 h (Oricell, USA). Stained samples were rinsed with distilled water and imaged.

### 2.5 Quantitative real–time PCR

Total RNA was extracted from ALCs using an RNA extraction kit (Vazyme). cDNA was synthesized from 1 μg RNA by using the HiScript II Q RT SuperMix for qPCR (Vazyme). Real–time RT–PCR was performed using ChamQ SYBR qPCR Master Mix (Vazyme) under a LightCycler® 96 System (Roche, Switzerland). The genes examined were Amelogenin (*Amelx*), Bone Morphogenetic Protein 4 (*Bmp4*), and Odontogenic Ameloblast–Associated Protein (*Odam*). *β–Actin* was used as an internal control. The primer sequences, designed and synthesized by GenScript (China), are listed in Supplementary Table S1.

### 2.6 RNA-seq and immunohistochemistry

To characterize the molecular response of ALCs to hyperthermia, cells were cultured under control conditions (37 °C for 7 days) or hyperthermic conditions (39 °C for 3 days followed by 37 °C for 4 days) and analyzed by RNA sequencing (Hangzhou Guangkeande Biotechnology, China). Differential expression was analyzed using DESeq2 with Benjamini–Hochberg false discovery rate correction. Genes with an adjusted *P* value <0.05 and an absolute log_2_ fold change >1 were considered differentially expressed. The top 200 differentially expressed genes ranked by statistical significance were used for pathway–enrichment analysis.

On the basis of the RNA-seq results, TRPV1 was prioritized for further investigation, and immunohistochemistry (IHC) was performed to assess TRPV1 expression in mouse ameloblasts. Tooth germs from postnatal day 2 (PN2) mice were fixed with 4% paraformaldehyde, decalcified in 10% EDTA, paraffin–embedded, and sectioned at 4 μm. After deparaffinization and antigen retrieval, sections were incubated with rabbit anti–mouse TRPV1 antibody (1:200; Abcam, UK). Negative–control sections were processed in parallel with omission of the primary antibody. Immunoreactivity was visualized using a DAB kit, counterstained with hematoxylin, and imaged using a digital slide scanner (3DHISTECH, Hungary).

### 2.7 Cell viability assay and inhibitor treatment

To identify a non–cytotoxic concentration range for AMG517, a selective TRPV1 antagonist (MCE, China), ALCs were seeded in 96–well plates (1 × 10^4^ cells/well) and exposed to 0, 0.1, 1, 2.5, 5, 10, 100 nM, or 1 μM AMG517 for 24 h. The TRPV1 inhibitory IC_50_ reported in the manufacturer’s official product information (0.5 nM) was used only as a pharmacological reference and was not determined in the present study. AMG517 powder was initially dissolved in a minimal volume of DMSO and subsequently diluted with serum–free DMEM/F-12 medium. The final DMSO concentration in the treatment medium was below 0.1% and was considered negligible under the experimental conditions. A separate DMSO–only control group was therefore not included. Cell viability was assessed using a CCK-8 kit (Beyotime, China), and absorbance at 450 nm was measured using a SpectraMax M3 microplate reader (Molecular Devices, USA). The CCK-8 assay was used solely to exclude potential cytotoxicity and not to determine the functional inhibitory potency of AMG517 against TRPV1.

### 2.8 Western blotting (WB) analysis

Total protein was extracted from ALCs and protein concentrations were determined using the BCA Protein Assay Kit (Beyotime, China). All steps followed the manufacturer’s instructions. Equal amounts of protein were separated by SDS–PAGE, transferred to PVDF membranes (Millipore, USA), and incubated with primary antibodies overnight at 4 °C, followed by HRP–conjugated secondary antibody (ABclonal, China). Primary antibodies included TRPV1 (1:2,000, Abcam, UK), AMELX (1:2,000, Abcam, UK), and ODAM (1:2,000, ABclonal, China). β–ACTIN (1:10,000, ABclonal, China) served as the loading control. Protein bands were visualized using an ECL chemiluminescence kit (Bio–Rad, China) and semi–quantitatively analyzed using ImageJ software.

### 2.9 shRNA and siRNA transfection

To investigate whether TRPV1 knockdown affects heat–induced inhibition of ALC mineralization, lentiviral particles carrying shRNA–TRPV1 or negative control (sh–NC) were obtained from GeneChem (Shanghai, China). The RNAi sequences are listed in Supplementary Table S2. ALCs were transduced according to the manufacturer’s protocol, and transfection efficiency was verified by qRT-PCR and Western blotting.

To avoid GFP fluorescence interference, TRPV1 was further silenced using siRNA–TRPV1 and si–NC (Bio–Rad, Shanghai, China; sequences listed in Supplementary Table S3). Transfection was performed using NB Transfection Reagent (Bio–Rad, China) according to the manufacturer’s instructions. TRPV1 silencing efficiency was confirmed at 36 and 48 h post–transfection.

### 2.10 Intracellular Ca^2+^ measurements (Fluo-4 AM) and intracellular ROS measurements (DCFH–DA)

Intracellular Ca^2+^ levels and ROS were measured using the Fluo-4 Calcium Assay Kit and the Reactive Oxygen Species Assay Kit (Beyotime, China), respectively. All steps followed the manufacturer’s instructions. ALCs exposed to different conditions (37 °C, 39 °C, 39 °C + si–NC, 39 °C + si-TRPV1, and 39 °C + AMG517) were incubated with Fluo-4 AM (10 μM) or DCFH–DA (10 μM), and analyzed by fluorescence microscopy (NIKON, Japan) and flow cytometry (SONY, Japan).

### 2.11 Mitochondrial calcium assay (Rhod–2 AM) and mitochondrial membrane potential detection (JC-1 Assay)

Mitochondrial Ca^2+^ levels and mitochondrial membrane potential (ΔΨm) were assessed using the Mitochondrial Calcium Assay Kit with Rhod–2 AM and the JC-1 Mitochondrial Membrane Potential Assay Kit (Beyotime, China), respectively. All steps were carried out according to the manufacturer’s instructions. ALCs were plated on glass–bottom confocal dishes (NEST, China), and fluorescence was observed under a laser confocal microscope (NIKON, Japan).

### 2.12 ATP and NAD^+^/NADH measurements

Mitochondrial ATP and NAD^+^/NADH levels were measured using commercial assay kits (Beyotime, China) following the manufacturer’s protocols. All steps followed the manufacturer’s instructions. ALCs were lysed, and ATP luminescence and NAD^+^/NADH ratios were determined using a SpectraMax M3 microplate reader (Molecular Devices, USA). ATP content was normalized to protein content and expressed as nmol/mg. The NAD^+^/NADH ratio was calculated according to the kit protocol.

### 2.13 Statistical analysis

Statistical analyses were performed using GraphPad Prism 10 (GraphPad Software, USA). ImageJ and FlowJo 10.8.1 were used for image and flow–cytometry analyses, respectively. Data are presented as the mean ± SD from three independent biological experiments, with each biological replicate averaged from three technical replicates where applicable. Normality was assessed using the Shapiro–Wilk test, and homogeneity of variance was evaluated using the Brown–Forsythe test. Data meeting these assumptions were compared using one-way ANOVA followed by Tukey’s multiple–comparison test. RNA-seq *P* values were adjusted using the Benjamini–Hochberg false discovery rate method. A two–sided *P* value <0.05 was considered statistically significant.

## 3 Results

### 3.1 Hyperthermia impairs mineralization in ALCs

Hyperthermic treatment significantly reduced the expression of the mineralization–related genes *Amelx*, *Bmp4*, and *Odam* (Figure 1A). Consistent with these molecular changes, ALP and ARS staining intensities were visibly reduced after hyperthermic exposure (Figures 1B,C). Because the 39 °C 3–day and 39 °C 5–day groups showed comparable reductions in the qRT-PCR readouts, the 39 °C 3–day condition was selected for subsequent mechanistic experiments.

**FIGURE 1:**
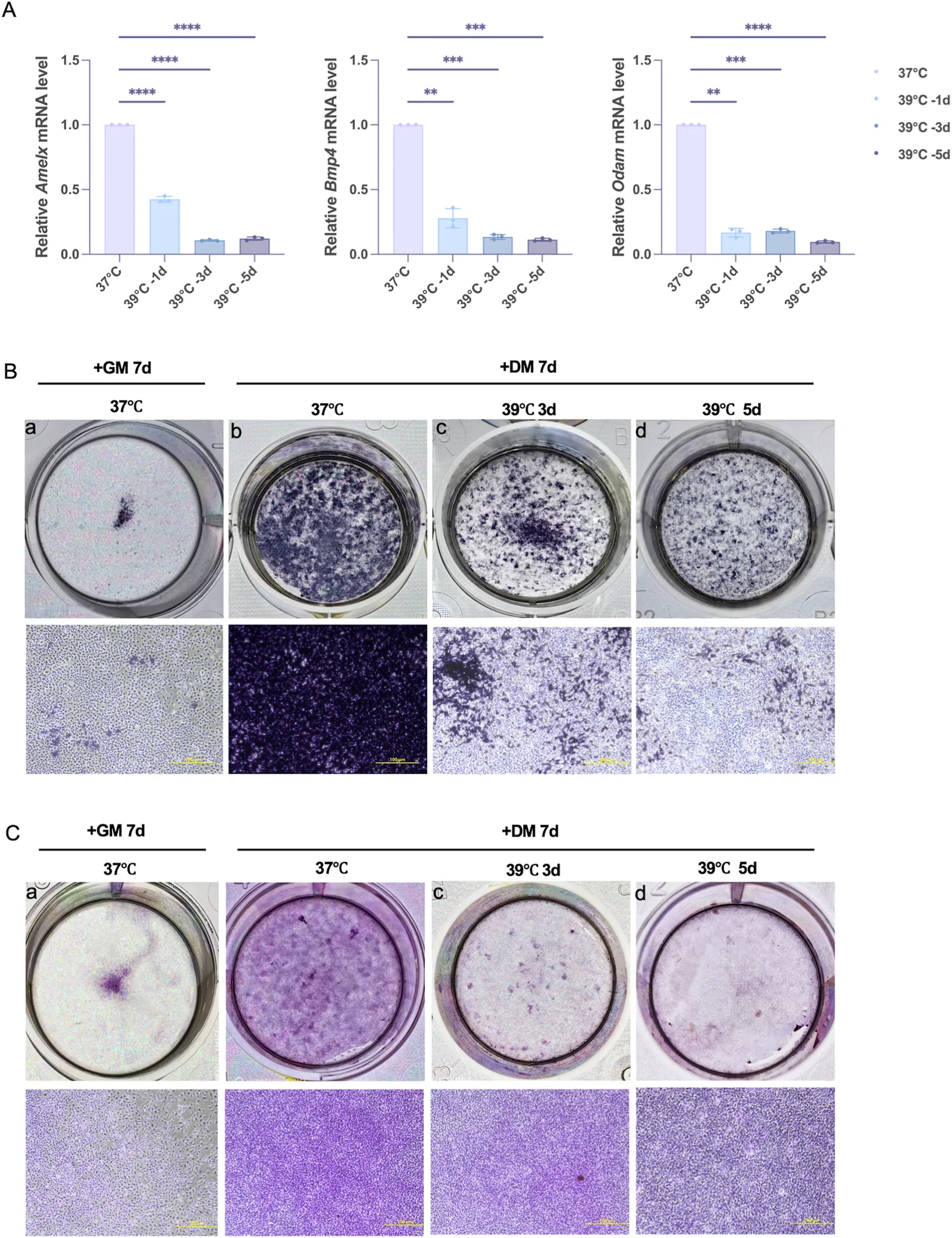
Effects of hyperthermia duration on ALC mineralization **(A)** qRT-PCR analysis after a total of 7 days of mineralization: 37 °C for 7 days; 39 °C for 1 day followed by 37 °C for 6 days; 39 °C for 3 days followed by 37 °C for 4 days; and 39 °C for 5 days followed by 37 °C for 2 days. *Amelx*, *Bmp4*, and *Odam* expression was normalized to β–Actin **(B,C)** Representative ALP staining on day 7 and ARS staining on day 21. a, unmineralized control at 37 °C; b, mineralized control at 37 °C; c, 39 °C for 3 days; d, 39 °C for 5 days. Scale bars are shown in the images. *n* = 3 independent biological experiments; *P < 0.05, **P < 0.01, ***P < 0.001, ****P < 0.0001; one-way ANOVA with Tukey’s multiple–comparison test.

### 3.2 Analysis of genes and signaling pathways in ALCs following hyperthermia

RNA-seq analysis identified 864 differentially expressed genes between the control (CTRL, 37 °C) and hyperthermia (HIGH, 39 °C) groups using an adjusted *P* value <0.05 and an absolute log_2_ fold change >1. Enrichment analyses indicated changes in pathways related to calcium transport, glycolysis/gluconeogenesis, heat responses, aerobic respiration, and mineralization (Figures 2A–F). The differential-expression statistics for *Trpv1* and other Ca^2+^-related or thermosensitive channel genes identified from the enriched GO terms are summarized in Supplementary Table S4. *Trpv1* was significantly upregulated under hyperthermic conditions (log_2_ fold change = 1.095; *P* = 0.00688; adjusted *P* = 0.02443) and was highlighted in the volcano plot (Figure 2E). IHC showed TRPV1–positive staining in the ameloblast region of PN2 mouse tooth germs, whereas the negative control showed no specific DAB signal (Figure 3A). These transcriptomic and tissue–expression data supported prioritizing TRPV1 for subsequent functional experiments.

**FIGURE 2:**
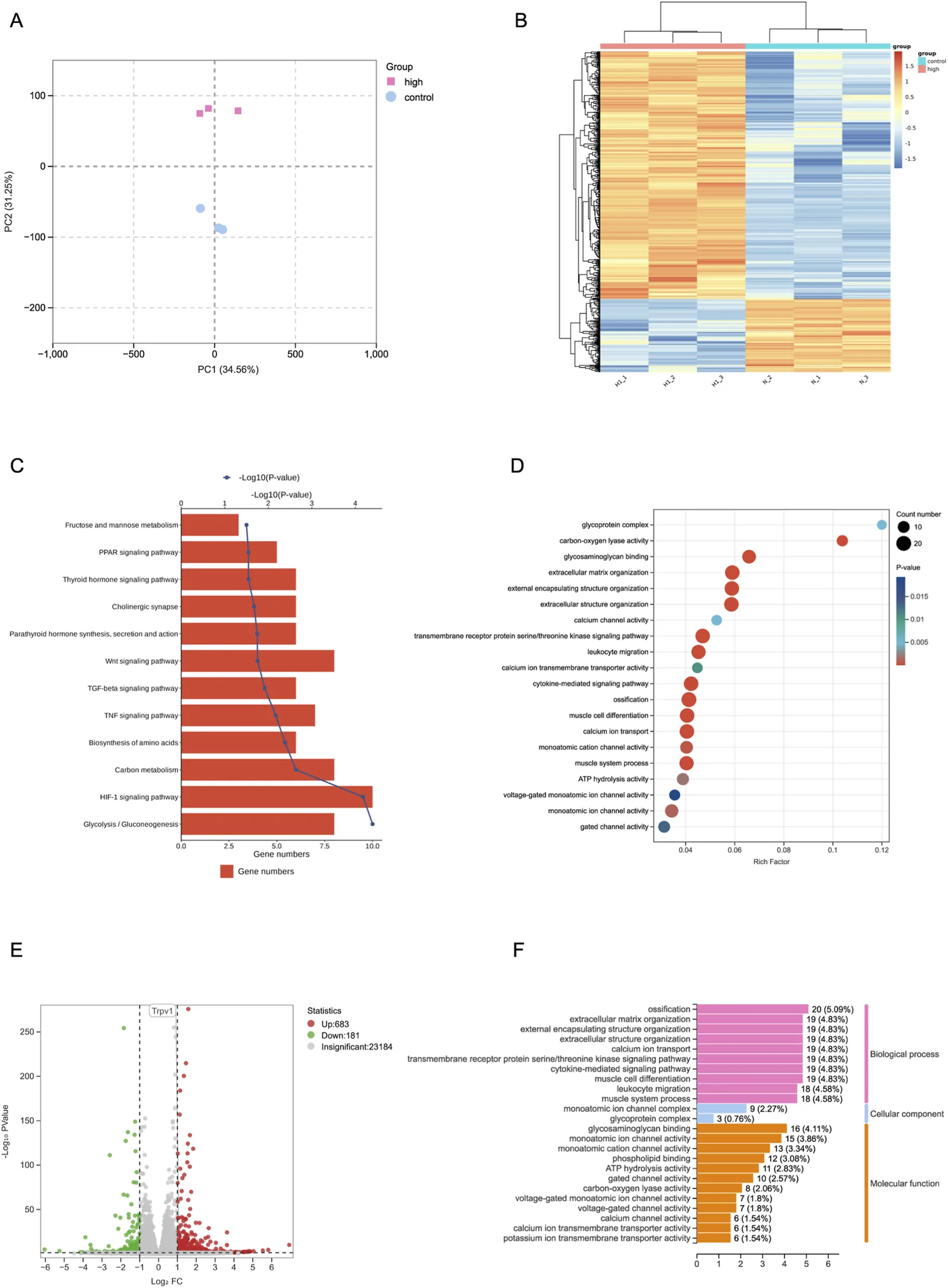
RNA-seq analysis of ALCs under control and hyperthermic conditions **(A)** PCA plot; PC1 and PC2 explain 34.56% and 31.25% of sample variance, respectively **(B)** Hierarchical–clustering heatmap of CTRL1–3 and HIGH1–3 samples **(C)** Top 12 enriched KEGG pathways **(D)** Significantly enriched GO terms **(E)** Volcano plot of differential expression under hyperthermia, with *Trpv1* labeled (log_2_ fold change = 1.095; adjusted *P* = 0.02443) **(F)** Top 24 enriched GO terms. Differential expression was defined as an adjusted *P* value <0.05 and an absolute log_2_ fold change >1.

**FIGURE 3:**
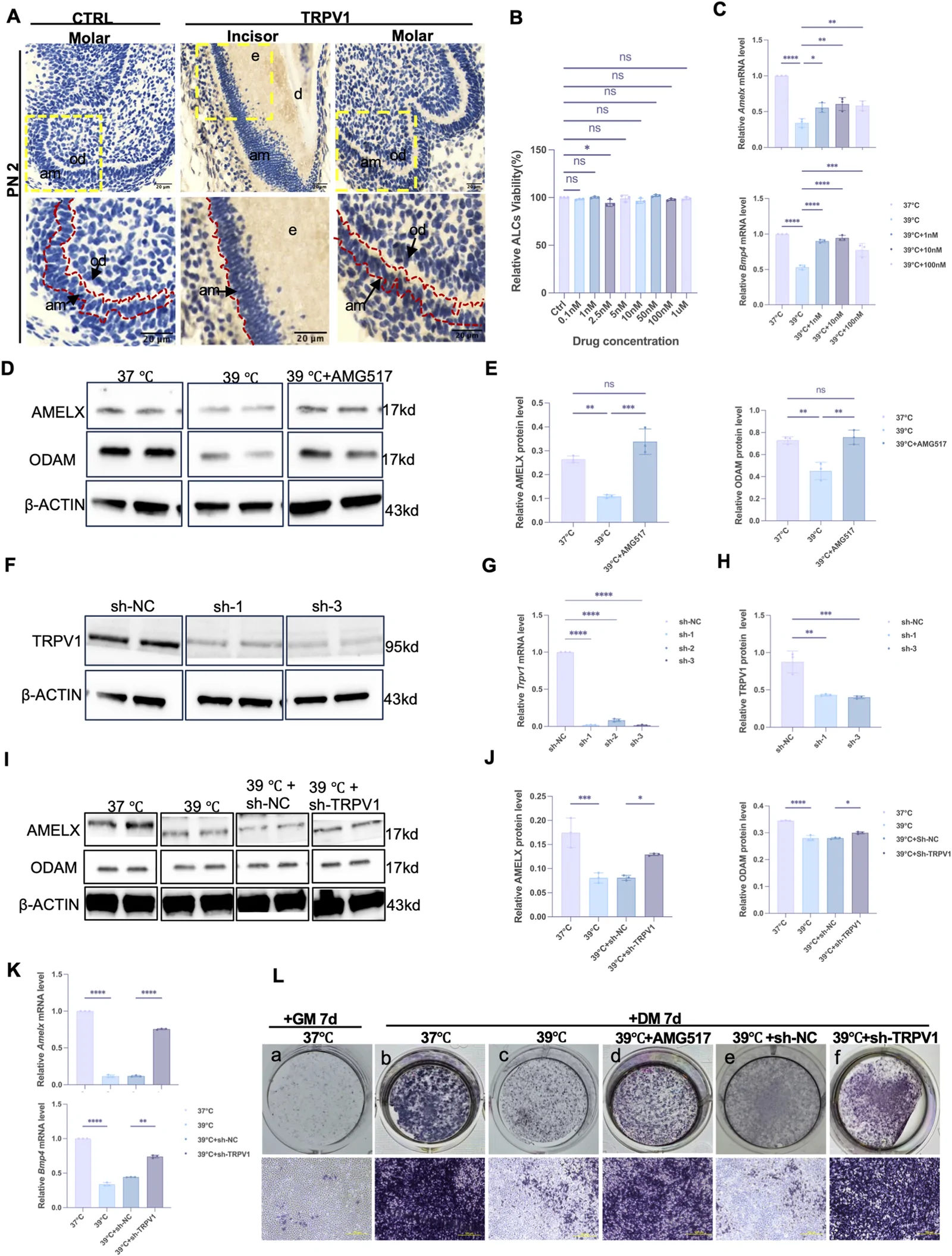
Pharmacological inhibition or knockdown of TRPV1 alleviates hyperthermia–associated mineralization impairment **(A)** TRPV1 IHC in PN2 mouse tooth germs, including a primary-antibody-omission negative control, enlarged views, and annotations of the ameloblast layer and positive–staining region. Yellow dashed boxes indicate the areas shown at higher magnification, whereas red dashed lines delineate the ameloblast distribution regions. am, ameloblasts; od, odontoblasts; d, dentin; e, enamel. Scale bar = 20 μm **(B)** ALC viability after 24 h of AMG517 exposure, assessed by CCK-8 assay to evaluate cytotoxicity **(C)** *Amelx* and *Bmp4* mRNA expression after treatment with 1, 10, or 100 nM AMG517 under hyperthermic conditions. 10 nM was selected for subsequent experiments based on the greatest and most consistent improvement **(D–E)** AMELX and ODAM protein abundance after treatment with 10 nM AMG517 **(F–H)** TRPV1 knockdown efficiency **(I–J)** AMELX and ODAM protein abundance after shRNA–mediated TRPV1 suppression **(K)** *Amelx* and *Bmp4* expression after TRPV1 suppression **(L)** Representative ALP staining. a, unmineralized control; b, mineralized control; c, 39 °C; d, 39 °C + 10 nM AMG517; e, 39 °C + sh–NC; f, 39 °C + sh–TRPV1. Scale bars are shown in the images. *n* = 3 independent biological experiments; *P < 0.05, **P < 0.01, ***P < 0.001, ****P < 0.0001; one-way ANOVA with Tukey’s multiple–comparison test.

### 3.3 Pharmacological inhibition or knockdown of TRPV1 alleviates hyperthermia–associated mineralization impairment in ALCs

To examine the functional involvement of TRPV1 in hyperthermia–associated mineralization impairment, we used AMG517, a selective TRPV1 antagonist (Garami et al., 2010), together with shRNA–mediated TRPV1 suppression.

CCK-8 analysis detected no cytotoxicity across the concentration range used in ALCs experiments (Figure 3B). A dose–response assessment of *Amelx* and *Bmp4* expression showed that 10 nM AMG517 produced a reproducible improvement under hyperthermic conditions, and this concentration was selected for subsequent experiments (Figure 3C).

Results of qRT-PCR and Western blotting showed a significant reduction in TRPV1 levels in the sh–TRPV1 group compared with the sh–NC group. The highest knockdown efficiency was observed in sh–3 (Figures 3F–H). Thus, a stable sh–TRPV1 cell line (sh–3) was selected for subsequent experiments.

Under 39 °C conditions, pharmacological inhibition or genetic suppression of TRPV1 increased *Amelx* and *Bmp4* mRNA levels (Figures 3C,K), increased AMELX and ODAM protein abundance (Figures 3D,E,I,J), and improved ALP staining (Figure 3L). These convergent pharmacological and genetic results support the involvement of TRPV1 in hyperthermia–associated mineralization impairment in ALCs.

### 3.4 TRPV1 inhibition or suppression improves hyperthermia–associated calcium, redox, and mitochondrial readouts in ALCs

To assess TRPV1 involvement in hyperthermia–associated calcium, redox, and mitochondrial responses, ALCs were transfected with siRNA–TRPV1 or si–NC. Efficient TRPV1 silencing was confirmed by qRT-PCR and Western blotting (Figures 4A–C). ALCs were then divided into five groups: 37 °C, 39 °C, 39 °C + AMG517, 39 °C + si–NC, and 39 °C + si-TRPV1.

**FIGURE 4:**
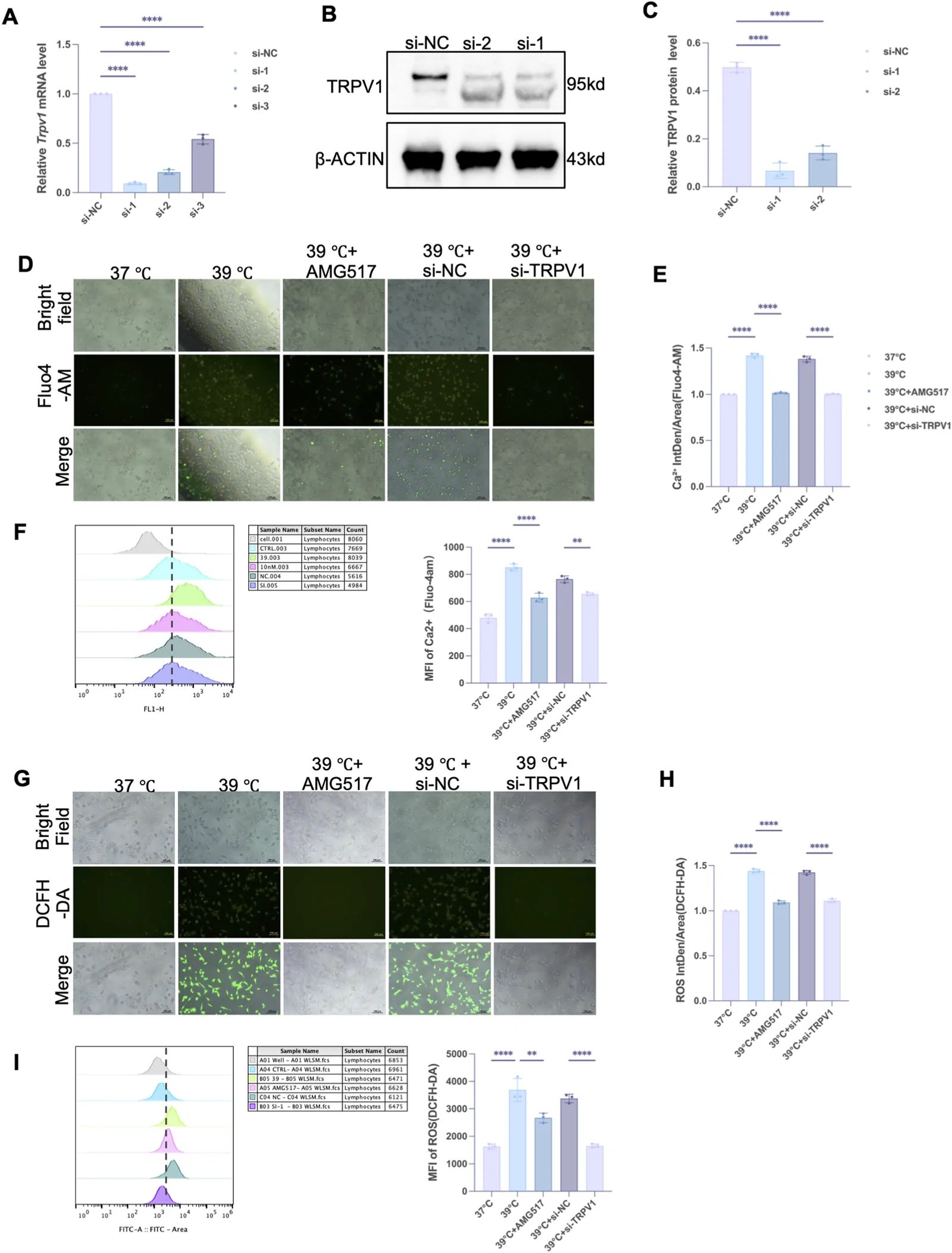
TRPV1 suppression reduces hyperthermia–associated calcium and ROS signals **(A–C)** qRT-PCR and Western blot analyses of siRNA–mediated TRPV1 suppression **(D)** Representative Fluo-4 AM fluorescence images **(E)** Image–based Fluo-4 AM quantification **(F)** Flow–cytometric Fluo-4 AM analysis and mean fluorescence intensity **(G)** Representative DCFH–DA fluorescence images **(H)** Image–based DCFH–DA quantification **(I)** Flow–cytometric DCFH–DA analysis and mean fluorescence intensity. Groups were 37 °C, 39 °C, 39 °C + 10 nM AMG517, 39 °C + si–NC, and 39 °C + si-TRPV1. Scale bar = 100 μm. *n* = 3 independent biological experiments; *P < 0.05, **P < 0.01, ***P < 0.001, ****P < 0.0001; one-way ANOVA with Tukey’s multiple–comparison test.

Fluo-4 AM analysis showed that 39 °C exposure increased intracellular Ca^2+^ fluorescence relative to the 37 °C group. AMG517 treatment or TRPV1 silencing reduced this increase (Figures 4D–F). Hyperthermia also increased intracellular ROS fluorescence, which was reduced by AMG517 or TRPV1 silencing (Figures 4G–I). These findings support an association between TRPV1 and hyperthermia-induced disturbances in calcium and redox homeostasis.

We next assessed mitochondrial Ca^2+^ signals, mitochondrial membrane potential, ATP content, and the NAD^+^/NADH ratio. Hyperthermia increased mitochondrial Ca^2+^ fluorescence and decreased the JC-1 aggregate/monomer ratio, ATP content, and NAD^+^/NADH ratio (Figures 5A–F). AMG517 treatment or TRPV1 silencing improved these readouts. Together, these findings support the involvement of TRPV1 in hyperthermia–associated mitochondrial dysfunction and suggest that altered calcium homeostasis may contribute to this response.

**FIGURE 5:**
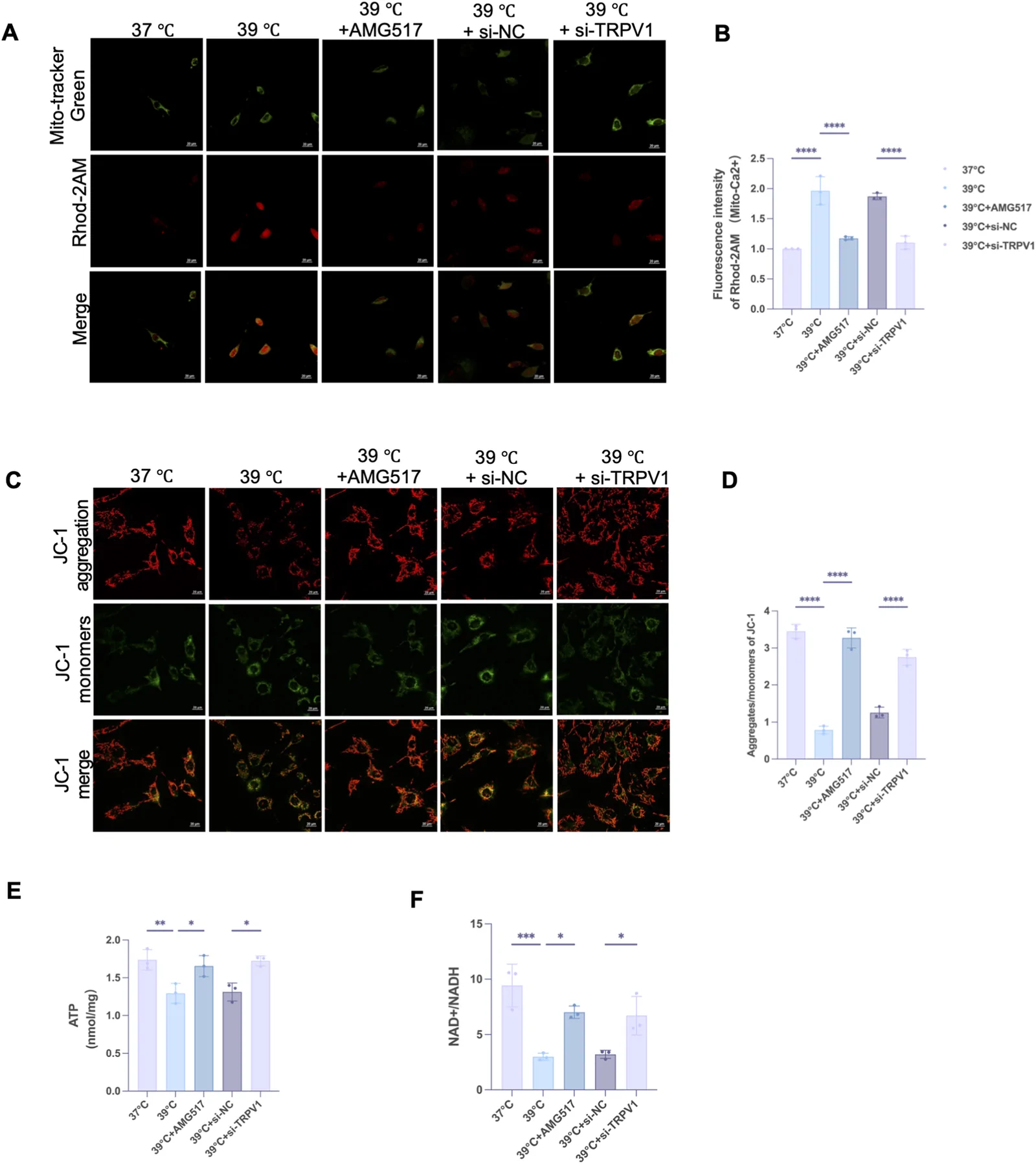
TRPV1 inhibition or suppression improves hyperthermia–associated mitochondrial readouts **(A–B)** Mitochondrial Ca^2+^ analysis using Rhod–2 AM and MitoTracker Green **(C–D)** JC-1 analysis of mitochondrial membrane potential **(E)** ATP content **(F)** NAD^+^/NADH ratio. Groups were 37 °C, 39 °C, 39 °C + 10 nM AMG517, 39 °C + si–NC, and 39 °C + si-TRPV1. Scale bar = 20 μm. *n* = 3 independent biological experiments; *P < 0.05, **P < 0.01, ***P < 0.001, ****P < 0.0001; one-way ANOVA with Tukey’s multiple–comparison test.

## 4 Discussion

Enamel hypomineralization has become increasingly prevalent among children (Ammar et al., 2025; Al–Nerabieah et al., 2025)Despite multiple proposed risk factors, including hyperthermia, its exact etiology and underlying mechanisms remain unclear (Malmberg et al., 2019). Given that fever is one of the most common reasons for outpatient visits and inpatient evaluation in children (Jayashree et al., 2024), its potential developmental consequences, including enamel hypomineralization, warrant further attention. Studies have reported that hyperthermic exposure during infancy can induce enamel hypomineralization (Weerheijm et al., 2003), but the related signaling pathways have not been defined. Meanwhile, hyperthermia can disrupt protein homeostasis and activate stress responses at the cellular level (Ahmed et al., 2020). Using an *in vitro* ALC model, we found that hyperthermia attenuated mineralization-associated readouts and that pharmacological inhibition or genetic suppression of TRPV1 partially alleviated these changes. These findings support the involvement of TRPV1 in the cellular response to hyperthermia in ALCs. Specifically, we first demonstrated that hyperthermia weakened the mineralization capacity of ALCs. After 3 days of hyperthermia exposure, the mRNA and protein levels of AMELX, BMP4, and ODAM, along with ALP activity and mineralization nodules, were markedly diminished. Our findings align with previous studies in mouse tooth germs (Ryynänen et al., 2014) and turpentine-induced fever in rats (Tung et al., 2006), demonstrating that hyperthermia impairs enamel mineralization (Teixeira et al., 2024). However, as prior research mainly focused on phenotypic changes without clarifying the underlying mechanisms, we conducted a more in-depth investigation.

Further RNA-seq analysis revealed that hyperthermia-associated DEGs were enriched in processes related to calcium transport, glycolysis/gluconeogenesis, and thermosensation, whereas downregulated DEGs were enriched in processes related to oxidative phosphorylation, aerobic respiration, and enamel mineralization. Ca^2+^ homeostasis is crucial for ameloblast differentiation and enamel mineralization (Eckstein et al., 2018). During maturation, ameloblasts highly express store-operated calcium entry (SOCE) components, including Stromal Interaction Molecule 1 (STIM1) and ORAI Calcium Release-Activated Calcium Modulator 1 (ORAI1). They sustain Ca^2+^ influx for hydroxyapatite deposition (Wang et al., 2014; Nurbaeva et al., 2015; Eckstein et al., 2019). Mitochondria serve as calcium buffers and energy producers (Costiniti et al., 2022) during mineralization. Through the mitochondrial calcium uniporter (MCU) pathway, they take up excess cytosolic Ca^2+^ to maintain calcium homeostasis and also enhance ATP production (Giorgi et al., 2018), thereby supporting enamel mineralization (D’Angelo et al., 2023). Dysfunction of these calcium-handling systems induces endoplasmic reticulum (ER) stress and mitochondrial damage, leading to mineralization defects.

This pattern is biologically relevant because glucose metabolism contributes to amelogenesis, and stage-specific metabolic shifts between glycolysis and oxidative phosphorylation participate in ameloblast phenotype modulation and enamel mineralization (Ida–Yonemochi, 2025; Arai et al., 2022).

The enrichment of calcium–transport and thermosensory pathways suggested the involvement of a thermosensitive cation channel. Among the major thermosensitive TRPV channels examined, *Trpv1* was significantly upregulated in the RNA-seq dataset, whereas other thermosensitive calcium channels (Diaz–Franulic et al., 2021) (such as *Trpv2*, *Trpv3*, *and Trpv4*) showed no significant differential expression and were not detected in significantly enriched gene sets. TRPV1 is a non–selective cation channel with substantial Ca^2+^ permeability (Benítez–Angeles et al., 2020; Zhang et al., 2018) and established heat sensitivity (Tominaga and Iwata, 2025). Beyond sensory neurons, TRPV1 is expressed in several non-neuronal cell types and participates in tissue stress responses (Bagood and Isseroff, 2021; Otto et al., 2020). IHC further demonstrated TRPV1 expression in the mouse ameloblast region (Figure 3A). These findings provided a biological and data–driven rationale for prioritizing TRPV1. TRPV1 expression has also been reported in the dental pulp of MIH-affected teeth (Rodd et al., 2007), whereas hypersensitivity is a common clinical feature of MIH (Linner et al., 2021). Although these observations concern dental pulp rather than ameloblasts, they further support the clinical relevance of TRPV1-related sensory signaling in hypomineralized teeth.

Pharmacological inhibition with AMG517 and genetic suppression of TRPV1 produced concordant improvements in mineralization–related molecular and staining readouts. The use of two independent intervention strategies reduces reliance on a single pharmacological observation and supports the functional involvement of TRPV1 in the ALC response to hyperthermia. Together, these findings identify TRPV1 as a candidate link between thermal stress (Xu et al., 2022) and ameloblast mineralization and extend its established roles in calcium signaling and mineralized–tissue biology (Hu et al., 2020; He et al., 2017).

The main validation strategy of this study was that hyperthermic stimulation increased cytosolic and mitochondrial Ca^2+^ signals, promoted ROS accumulation and mitochondrial membrane depolarization, and impaired mitochondrial bioenergetic function, as reflected by decreased ATP content and a reduced NAD^+^/NADH ratio. These alterations were accompanied by impaired mineralization-related readouts and may have reduced the energy available for mineralization, ultimately contributing to impaired mineralization in ALCs (Figure 6). However, AMG517 treatment or TRPV1 silencing ameliorated these abnormal responses. Together, the pharmacological and genetic interventions demonstrate that TRPV1 participates in mediating the coordinated calcium–mitochondrial response of ALCs to hyperthermia. Previous studies have shown that TRPV1-mediated Ca^2+^ transfer to mitochondria can disturb intracellular calcium homeostasis (Tessier et al., 2023; Xu et al., 2020). Mitochondrial Ca^2+^ overload may subsequently enhance ROS generation (Adam–Vizi et al., 2010), reduce mitochondrial membrane potential (Sun et al., 2014), and impair mitochondrial metabolism and ATP production (Giorgi et al., 2018; Brookes et al., 2004). By integrating these mitochondrial functional indicators with mineralization-related protein expression and staining outcomes, the present study extends the existing evidence to ameloblast-lineage cells and supports TRPV1 as an important molecular link connecting hyperthermic stress, calcium–mitochondrial dyshomeostasis, and impaired ALC mineralization. A principal contribution of this study is the integration of transcriptomic screening, tissue localization, pharmacological inhibition (Gavva et al., 2007), genetic suppression, calcium and ROS measurements, mitochondrial functional readouts, and mineralization assays within a single experimental framework. This multilevel design provides convergent evidence that TRPV1 participates in the ALC response to hyperthermia and offers a testable link between environmental temperature, organelle function, and mineralization.

**FIGURE 6:**
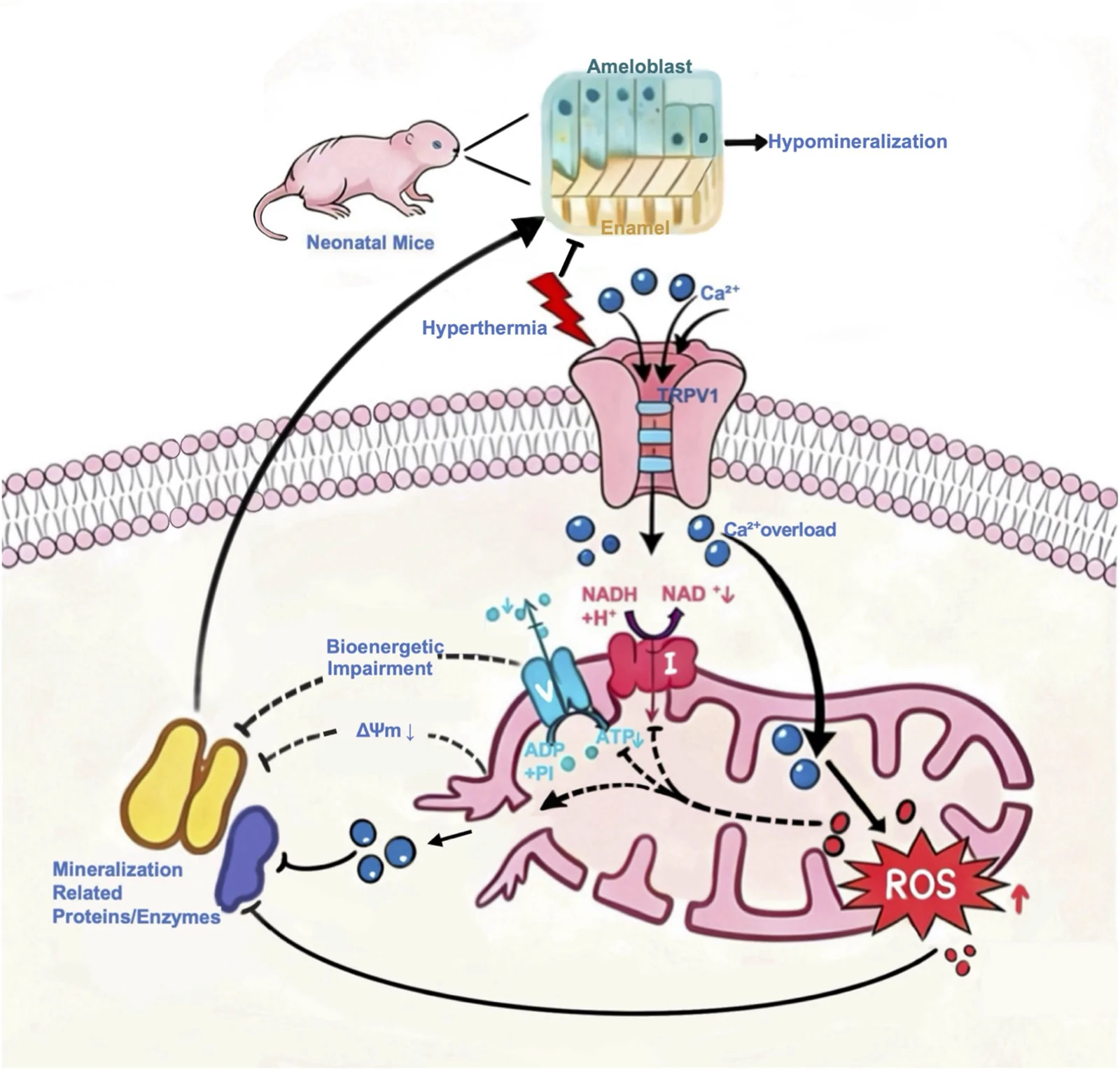
Working model of the TRPV1–associated response to hyperthermia in ALCs. The model summarizes the associations among the indicators observed in this study: hyperthermia triggers responses involving TRPV1, leading to increased cytosolic and mitochondrial Ca^2+^ signals, ROS accumulation, a reduced JC-1 aggregate/monomer ratio, and decreased ATP content and NAD^+^/NADH ratio, which may suggest hyperthermia-induced opening of the mitochondrial permeability transition pore (mPTP) and impairment of respiratory–chain complexes I/V. These changes were accompanied by reduced *Amelx*, *Bmp4*, and *Odam* mRNA expression, decreased AMELX and ODAM protein abundance, and weaker ALP and ARS staining, ultimately resulting in impaired cellular mineralization. AMG517 treatment or TRPV1 suppression partially reversed these abnormal readouts. This model provides a basis for further mechanistic investigation and *in vivo* validation. (Dashed arrows denote proposed associations rather than a proven causal sequence).

The present study still has several limitations. First, all functional experiments were performed using only a single ameloblast–lineage cell line. Second, immunohistochemistry only confirmed TRPV1 expression in mouse ameloblasts but could not verify its physiological function *in vivo*. In addition, whether calcium overload is an essential component of this pathway and whether other channels also participate in this response remain to be investigated. Therefore, in future studies, we will conduct additional intervention experiments using calcium chelators (Konečný et al., 2024), such as BAPTA–AM, and mitochondrial calcium uptake inhibitors, and use conditional gene–edited animal models to verify whether this regulatory pathway is functionally operative during enamel development *in vivo*.

## 5 Conclusion

In conclusion, this study shows that hyperthermia impairs mineralization–related readouts in cultured ALCs and is accompanied by increased calcium and ROS signals and altered mitochondrial functional indicators. Pharmacological inhibition and genetic suppression of TRPV1 alleviated these changes, supporting TRPV1 as a contributor to the cellular response to hyperthermia. These findings establish a testable TRPV1–associated calcium–mitochondrial framework for enamel research. Future *in vivo* studies will further validate this framework and establish its potential value in guiding disease prevention and clinical treatment.

## Data Availability

The raw RNA‐sequencing datasets generated for this study are available from the corresponding author upon reasonable request. All other datasets supporting the conclusions of this article are included in the article and its supplementary material.
