# Supplementary material for: TRPV1 mediates mitochondrial dysfunction in hyperthermia–induced ameloblast mineralization defects
Source: Front Cell Dev Biol. 2026 Sep 17;14:1903038. doi: 10.3389/fcell.2026.1903038 (PMC13627420; doi:10.3389/fcell.2026.1903038)
Supplement: Supplementary file 1 [file DataSheet1.pdf]

TRPV1 Mediates Mitochondrial Damage in Hyperthermia-Induced Ameloblast Mineralization Defects

Jingyi Li[1], Jing Fu[1], Yiwen Pan[1], Huiwen Zheng[1]* and Xianghui Xing[1]*

[1] Nanjing Stomatological Hospital, Affiliated Hospital of Medical School, Institute of Stomatology, Nanjing University, Nanjing, China

***Correspondence:**
Huiwen Zheng

E-mail: 781063817@qq.com

Xianghui Xing

E-mail: dr.xing@nju.edu.cn

Supplemental Tables

Gene	Gene Primer（5'→3'）
Amelx-F'	CCCCAGTCACCTCTGCATC
Amelx-R'	GCTGCATGGAGAACAGTGG
Bmp4-F'	TTCCTGGTAACCGAATGCTGA
Bmp4- R'	CCTGAATCTCGGCGACTTTTT
Odam-F'	GTCACATCCTCACCACAGCA
Odam- R'	GAGTTTCTGGAGCTGTGCCT
Trpv1-F'	ACCACGGCTGCTTACTATCG
Trpv1-R'	TCCCCAACGGTGTTATTCAG
β-Actin-F'	ATGACCCAAGCCGAGAAGG
β-Actin- R'	CGGCCAAGTCTTAGAGTTGTTG

Table S1. The primers for *Amelx*，*Bmp4*，*Odam*，*Trpv1*，*β-Actin*.

RNAi sequence

Label	Target Seq
LV-Trpv1-RNAi(PSC124624-1)	CCATCCCTCAAGAGTTTGTTT
LV-Trpv1-RNAi(PSC124625-1)	CCCACACTGAAGCTAGAAGAA
LV-Trpv1-RNAi(PSC124626-1)	GCCATGCTCAATCTGCACAAT

Vector Information

	Information
Vector Name	GV493
Element Order	hU6-MCS-CBh-gcGFP-IRES-puromycin
Cloning Sites	AgeI, EcoRI
Control Number	CON313
Control Insert Sequence	TTCTCCGAACGTGTCACGT

Table S2. The information of sh-RNA.

siRNA sequence

Label	Sequence(5'→3')
M-siTrpv1-1(1909)	AUGCUCUACUACACCCGAGGATT<br>UCCUCGGGUGUAGUAGAGCAUTT
M-siTrpv1-2(2204)	CCGAGAACUAUGACUUCAAGGTT<br>CCUUGAAGUCAUAGUUCUCGGTT
M-siTrpv1-3(1532)	GGGACAGAUUUGUCAAGCGCATT<br>UGCGCUUGACAAAUCUGUCCCTT

Table S3. The information of si-RNA.

Table S4 DEG table including other Ca^{2+}-related or thermosensitive channel genes

Gene	Ensembl ID	Mean count, hyperthermia	Mean count, control	log_2FC	*P* value	FDR-adjusted *P* value	Regulation
Slc24a1	ENSMUSG00000034452	133.00	38.00	1.800	4.45E-15	1.06E-13	Upregulated
Slc24a5	ENSMUSG00000035183	77.33	33.33	1.221	5.80E-06	0.00004	Upregulated
Cacna2d3	ENSMUSG00000021991	18.67	3.33	2.492	0.00024	0.00127	Upregulated
P2rx5	ENSMUSG00000005950	45.00	18.00	1.326	0.00021	0.00113	Upregulated
Grin3a	ENSMUSG00000039579	16.33	3.67	2.185	0.00206	0.00870	Upregulated
**Trpv1**	**ENSMUSG00000005952**	**32.67**	**15.33**	**1.095**	**0.00688**	**0.02443**	**Upregulated**
Trpc6	ENSMUSG00000031997	17.67	6.00	1.555	0.01510	0.04705	Upregulated
Cngb1	ENSMUSG00000031789	33.33	6.67	2.329	2.72E-06	0.00002	Upregulated
Chrnb2	ENSMUSG00000027950	42.67	19.67	1.111	0.00385	0.01487	Upregulated
Jph2	ENSMUSG00000017817	64.00	30.33	1.074	0.00024	0.00128	Upregulated
Casq1	ENSMUSG00000007122	25.33	10.00	1.334	0.00532	0.01956	Upregulated
Plcb1	ENSMUSG00000051177	2.67	11.33	-2.129	0.01143	0.03734	Downregulated

Bnip3	ENSMUSG00000078566	3113.33	975.67	1.663	1.64E-134	1.11E-131	Upregulated
Pygm	ENSMUSG00000032648	47.00	11.00	2.098	2.87E-07	2.72E-06	Upregulated
Htr2b	ENSMUSG00000026228	66.00	31.33	1.062	0.00048	0.00239	Upregulated
Gp1ba	ENSMUSG00000050675	44.33	21.00	1.075	0.00177	0.00759	Upregulated
Spp1	ENSMUSG00000029304	117.33	702.33	-2.582	6.97E-112	3.31E-109	Downregulated
Cxcl10	ENSMUSG00000034855	51.00	222.67	-2.132	6.24E-29	3.88E-27	Downregulated
Calcb	ENSMUSG00000030666	5.67	83.67	-3.879	2.11E-19	7.37E-18	Downregulated
Hap1	ENSMUSG00000006930	58.33	155.00	-1.424	3.68E-11	5.92E-10	Downregulated
Ccl5	ENSMUSG00000035042	4.33	16.33	-1.949	0.00475	0.01778	Downregulated
Gas6	ENSMUSG00000031451	5975.67	2000.00	1.578	1.35E-276	7.03E-273	Upregulated
Gpr35	ENSMUSG00000026271	82.00	0.67	6.935	8.08E-11	1.25E-09	Upregulated
Adrb2	ENSMUSG00000045730	49.00	101.67	-1.069	7.71E-06	0.00006	Downregulated
Adra1b	ENSMUSG00000050541	2.00	11.33	-2.479	0.00734	0.02577	Downregulated
Cxcl3	ENSMUSG00000029379	1.33	8.67	-2.661	0.01241	0.03992	Downregulated
Ghrl	ENSMUSG00000064177	37.67	15.00	1.321	0.00140	0.00621	Upregulated
Gng3	ENSMUSG00000071658	36.00	14.67	1.304	0.00129	0.00575	Upregulated
Ptgfr	ENSMUSG00000028036	71.67	21.67	1.722	4.46E-08	4.77E-07	Upregulated

Table S4 DEG table including other Ca^{2+}-related or thermosensitive channel genes
